# Partial Reconstruction of the Ergot Alkaloid Pathway by Heterologous Gene Expression in *Aspergillus nidulans*

**DOI:** 10.3390/toxins5020445

**Published:** 2013-02-22

**Authors:** Katy L. Ryan, Christopher T. Moore, Daniel G. Panaccione

**Affiliations:** Genetics and Developmental Biology Program, Division of Plant and Soil Sciences, West Virginia University, Morgantown, WV 26506, USA; E-Mails: kryan10@mix.wvu.edu (K.L.R.); cmoore6@mix.wvu.edu (C.T.M.)

**Keywords:** ergot alkaloids, mycotoxins, *Aspergillus fumigatus*, gene cluster, chanoclavine-I

## Abstract

Ergot alkaloids are pharmaceutically and agriculturally important secondary metabolites produced by several species of fungi. Ergot alkaloid pathways vary among different fungal lineages, but the pathway intermediate chanoclavine-I is evolutionarily conserved among ergot alkaloid producers. At least four genes, *dmaW*, *easF*, *easE*, and *easC*, are necessary for pathway steps prior to chanoclavine-I; however, the sufficiency of these genes for chanoclavine-I synthesis has not been established. A fragment of genomic DNA containing *dmaW*, *easF*, *easE*, and *easC* was amplified from the human-pathogenic, ergot alkaloid-producing fungus *Aspergillus* *fumigatus* and transformed into *Aspergillus* *nidulans*, a model fungus that does not contain any of the ergot alkaloid synthesis genes. HPLC and LC-MS analyses demonstrated that transformed *A. nidulans* strains produced chanoclavine-I and an earlier pathway intermediate. *Aspergillus* *nidulans* transformants containing *dmaW*, *easF*, and either *easE* or *easC* did not produce chanoclavine-I but did produce an early pathway intermediate and, in the case of the *easC* transformant, an additional ergot alkaloid-like compound. We conclude that *dmaW*, *easF*, *easE*, and *easC* are sufficient for the synthesis of chanoclavine-I in *A. nidulans* and expressing ergot alkaloid pathway genes in *A. nidulans* provides a novel approach to understanding the early steps in ergot alkaloid synthesis.

## 1. Introduction

**Figure 1:**
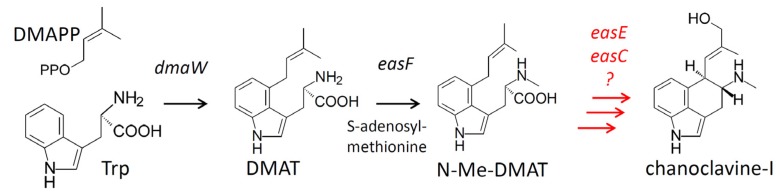
Early steps of the ergot alkaloid pathway. Genes controlling specific steps are listed beside arrows. Abbreviations: DMAPP = dimethylallyldiphosphate; Trp = tryptophan; DMAT = dimethylallyltryptophan. Unresolved steps are indicated in red.

Ergot alkaloids are tryptophan-derived mycotoxins produced by several different fungi and have proven to be both pharmaceutically and agriculturally important. Several *Claviceps*, *Neotyphodium*, and *Epichloë* species of the order Hypocreales produce a variety of complex ergot alkaloids [1,2]. *Aspergillus* *fumigatus* and *Penicillium commune* of the order Eurotiales also are capable of ergot alkaloid synthesis; however, their ergot alkaloid profiles differ considerably from those of the Hypocrealean ergot alkaloid producers [2,3]. Ergot alkaloid-producing fungi contain an ergot alkaloid synthesis (*eas*) gene cluster with a set of core genes that is well conserved [1,2,3,4]. The *eas* clusters of the various ergot alkaloid producers also contain variable, lineage-specific genes that result in the formation of different pathway products among lineages [2,4]. The steps of ergot alkaloid synthesis leading to the formation of chanoclavine-I (the earliest ergot alkaloid pathway biosynthetic intermediate observed accumulating in most producers) (Figure 1) are believed to be evolutionarily conserved among the different fungi that produce ergot alkaloids [1,2,3,4]; however, the identity and catalytic activities of all the genes required for chanoclavine-I synthesis have not yet been determined. 

Based on the proposed biochemical scheme of Kozikowski *et al*. [5], the synthesis of chanoclavine-I occurs in at least five steps. Gene knockout and heterologous expression studies have demonstrated that at least four genes, *dmaW*, *easF*, *easE*, and *easC*, are required prior to chanoclavine-I [6,7,8,9]. However, it is currently unknown whether these genes are sufficient to synthesize chanoclavine-I. DMAT synthase, the product of *dmaW*, catalyzes the initial prenylation reaction to yield dimethylallyltryptophan (DMAT) [6,10,11], and *easF* encodes a methyltransferase that *N*-methylates DMAT to form the second intermediate, *N*-Me-DMAT [7]. The order of activity and function of EasE and EasC have not been established, though gene knockout studies indicate these enzymes are necessary for the conversion of *N*-Me-DMAT to chanoclavine-I [8,9]. Based on their sequences, *easE* encodes an oxidoreductase and *easC* encodes a catalase. Involvement of additional enzymes cannot be excluded because the synthesis of chanoclavine-I requires two two-electron oxidations, and EasE is the only oxidoreductase among the four required genes. However, the *eas* clusters in the diverse fungi that produce ergot alkaloids do not contain any additional conserved oxidoreductase genes for which a function has not been assigned. Thus, the question of sufficiency of the four required genes is unresolved. 

*Aspergillus fumigatus* is a common saprophytic fungus and potentially fatal pathogen in immunocompromised individuals [12]. Ergot alkaloid synthesis genes are clustered in the *A. fumigatus* genome [10,11] and are expressed during asexual reproduction [13,14]. *Aspergillus* *nidulans*, a model fungus that is closely related to *A*. *fumigatus*, does not contain the ergot alkaloid gene cluster nor does it produce ergot alkaloids. The primary objective of this study was to test if *dmaW*, *easF*, *easC*, and *easE* are sufficient to direct chanoclavine-I synthesis in *A*. *nidulans*. We used a novel approach to test the sufficiency of the genes by transforming *A*. *nidulans* with *dmaW*, *easF*, *easC*, and *easE*, or subsets of those genes, from *A*. *fumigatus* under the control of their native promoters. 

## 2. Results

### 2.1. Engineering of Chanoclavine-I Producing Strains of *A. nidulans*

**Figure 2:**
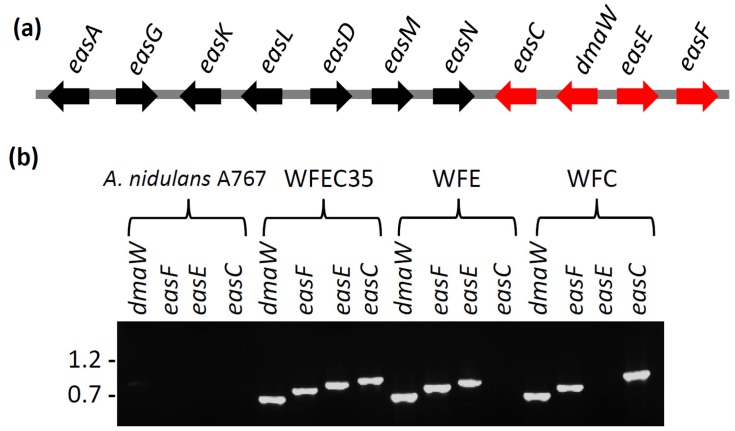
Ergot alkaloid synthesis genes. (**a**) The ergot alkaloid gene cluster of *A. fumigatus*. Genes expressed in *A. nidulans* in this study are shown in red; (**b**) PCR verification of the presence or absence of *dmaW*, *easF*, *easE*, and *easC* from wild type and mutant strains. Each gene was independently amplified from internal primer binding sites. Relative mobility of relevant *Bst*EII-digested bacteriophage lambda fragments (sizes in kb) is indicated to the left of the gel.

An 8.8-kb fragment of *A. fumigatus* DNA containing the genes *dmaW*, *easF*, *easE*, and *easC* was successfully amplified by PCR and integrated into the genome of two *A. nidulans* transformants, yielding strains WFEC33 and WFEC35. The presence of each of the four *A. fumigatus* genes in the WFEC strains was verified by PCR primed from internal gene primer-annealing sites (Figure 2). The recipient strain *A. nidulans* A767 did not contain any of the four genes. The absence of the ergot alkaloid gene cluster in *A. nidulans* was further supported by the lack of sequences with an E value less than 10 in a BLASTn search between the *A. fumigatus* *dmaW*, *easF*, *easE*, and *easC* cluster sequence with the genome of *A. nidulans* FGSC A4.

**Figure 3:**
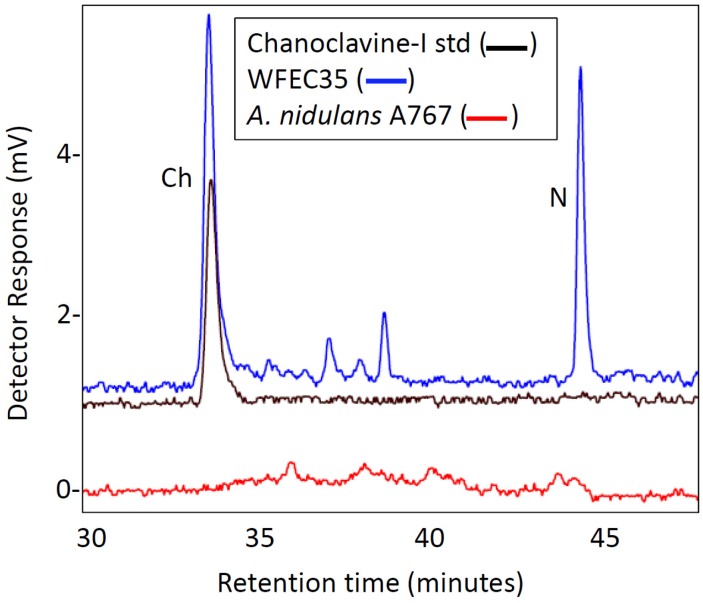
HPLC analyses of *A. nidulans* strain WFEC35 (transformed with *dmaW*, *easF*, *easE*, and *easC*), *A. nidulans* FGSC A767, and chanoclavine-I standard. Ch, chanoclavine-I; N, *N*-Me-DMAT. Fluorescence properties were observed with an excitation wavelength of 272 nm and an emission wavelength of 372 nm.

**Figure 4:**
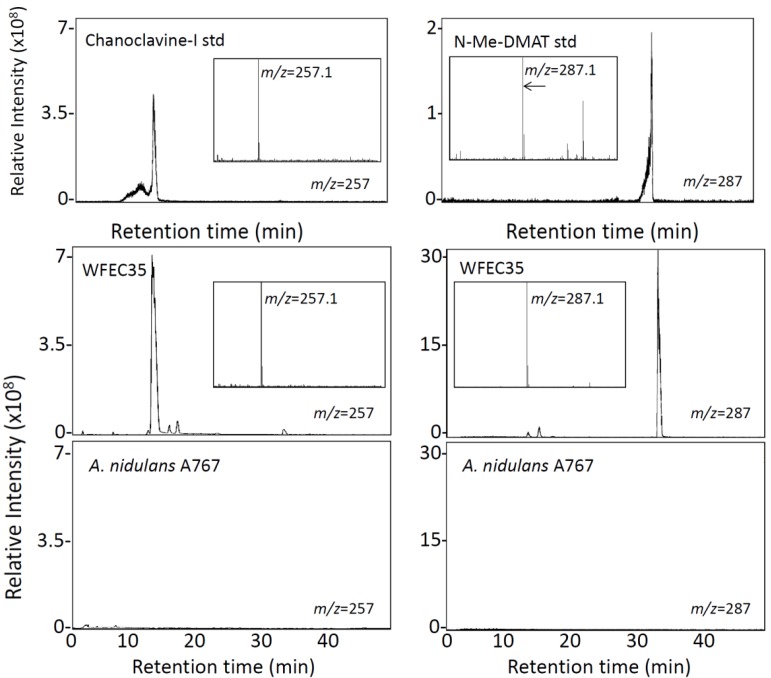
LC-MS analysis of *A. nidulans* extracts. Chromatograms display presence and elution time of molecules with an *m/z* value of 257 ± 0.5 (left column) or 287 ± 0.5 (right column). Chromatograms of *A. nidulans* strains A767 and WFEC35 were normalized to the intensity of the highest peak in the WFEC35 trace for each analyte. The *N*-Me-DMAT standard chromatogram is displayed at a higher sensitivity because of paucity of material available. Inserts show the mass spectrum of the major peak in each chromatogram.

Both WFEC strains and *A. nidulans* A767 were analyzed by HPLC with fluorescence detection to investigate the presence of ergot alkaloids. Transformed strains accumulated an analyte that co-eluted with chanoclavine-I at approximately 33 min, whereas *A. nidulans* A767 did not (Figure 3). Extracts of WFEC35 and *A. nidulans* A767 also were analyzed by electrospray ionization (ESI) LC-MS in positive mode. Again WFEC35 contained a peak that co-eluted with chanoclavine-I standard. The analyte had an *m/z* of 257.1 consistent with the [M + H]^+^ of chanoclavine-I (Figure 4). HPLC with fluorescence detection of mutant strain WFEC35 also revealed the presence of the early pathway intermediate *N*-Me-DMAT which eluted at approximately 44 min (Figure 3). The identity of this analyte as *N*-Me-DMAT was supported by an ion of *m/z* 287.1 in ESI LC-MS analyses (corresponding to [*N*-Me-DMAT + H]^+^) and by co-elution with authentic standard (Figure 4). 

Conidiating cultures produced modest quantities of chanoclavine-I. *Aspergillus nidulans* strain WFEC35 produced approximately 0.3 fg chanoclavine-I per conidium, or about 3 ng chanoclavine-I per mm^2^ of culture surface area and comparable quantities of *N*-Me-DMAT. Strain WFEC33 produced lesser quantities of chanoclavine-I and did not accumulate measurable quantities of *N*-Me-DMAT. 

### 2.2. Engineering of *A. nidulans* Strains Expressing Subsets of Early Pathway Genes

**Figure 5:**
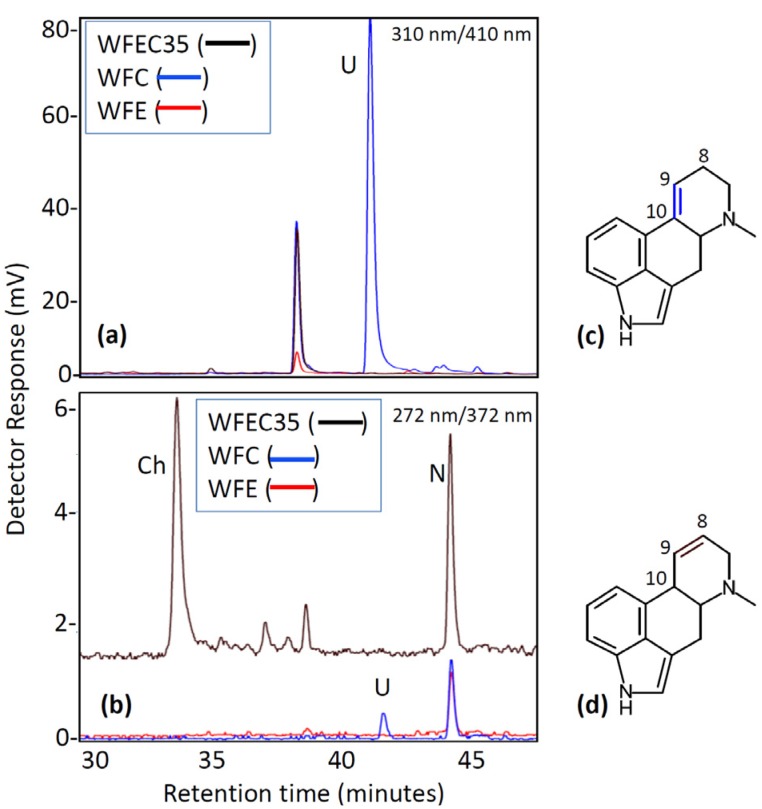
Fluorescence HPLC analysis of WFC and WFE mutant cultures. (**a**) Chromatogram with excitation at 310 nm and emission at 410 nm; (**b**) Chromatogram with excitation at 272 nm and emission at 372 nm; (**c**) Generic ergoline ring system with double bond between carbons 9 and 10; (**d**) Generic ergoline ring system with double bond between carbons 8 and 9. Ch, chanoclavine-I; N, *N*-Me-DMAT; U, Unknown unique to strain WFC.

Two additional mutant strains of *A. nidulans* were generated by transformation with subsets of three of the four ergot alkaloid cluster genes: strains WFC (containing *dmaW*, *easF*, and *easC*) and WFE (containing *dmaW*, *easF*, and *easE*). The presence of each gene in the genomes of the different mutants was verified by PCR (Figure 2). Chanoclavine-I was not detectable by HPLC (Figure 5) or ESI LC-MS (data not shown) in either strain. Expression of *dmaW* and *easF* from the inserted construct in both strains was validated by the accumulation of the early pathway intermediate, *N*-Me-DMAT, which eluted from the column at approximately 44 min (Figure 5). In addition, WFC cultures accumulated a novel molecule with an elution time of 41 min and that had an *m/z* value of 259.0 in ESI LC-MS analyses. The unidentified metabolite displayed a higher fluorescence at excitation and emission wavelengths of 310 nm and 410 nm, respectively, than at 272 nm excitation and 372 nm emission settings (Figure 5). Ergot alkaloids with a double bond between carbons 9 and 10 of the ergot alkaloid ring system fluoresce stronger at the 310 nm (excitation) and 410 nm (emission) setting compared to their fluorescence at the 272 nm/372 nm setting; conversely, ergot alkaloids with a 8,9 double bond or those lacking a double bond at either of those positions fluoresce stronger at the 272 nm/372 nm setting [15]. The novel alkaloid was not produced in sufficient quantities to allow the acquisition of additional structural data at this time. 

## 3. Discussion

Our results describe the first transfer of ergot alkaloid biosynthetic capability to a previously non-producing fungus. These data also indicate that *dmaW*, *easF*, *easE*, and *easC* are sufficient for *A. nidulans* to synthesize chanoclavine-I. Since the pathway leading to the formation of chanoclavine-I appears to be evolutionarily conserved, the knowledge gained from this study will not only apply to *A. fumigatus* but theoretically to all ergot alkaloid producers. Despite the large amount of research conducted on ergot alkaloid synthesis, the formation of chanoclavine-I has yet to be completely understood. Only two steps in this evolutionarily conserved portion of the pathway have been completely characterized—those controlled by *dmaW* and *easF* [6,7,10,11]. After the formation of *N*-Me-DMAT, the order of activity and catalytic functions of enzymes is not clear. Our data indicate that EasE and EasC individually are necessary and together are sufficient to convert *N*-Me-DMAT to chanoclavine-I. Nonetheless, the observations in this study do not rule out the possibility that *A. nidulans* naturally possesses an enzyme that also contributes to the formation of chanoclavine-I from *N*-Me-DMAT. Despite the fact that all known ergot alkaloid genes are clustered in *A. fumigatus* and other ergot alkaloid-producing fungi [1,2,3,4,15], we cannot presently exclude the possibility that a required gene in the pathway may be located elsewhere in the genome and common to non-producing fungi. 

Transformations with subsets of three of the four studied genes, combining alternately *easC* or *easE* with the combination of *dmaW* and *easF*, did not yield strains capable of producing chanoclavine-I. These results support previous knockout data indicating that both *easE* and *easC* are required for chanoclavine-I synthesis from *N*-Me-DMAT [9]. A metabolite of undetermined structure accumulated to relatively high levels in the WFC transformant; this metabolite was not detectable in extracts of the *A. nidulans* WFE strain. Improved strains or altered growth conditions will be necessary to increase yield of the compound for the purpose of collecting adequate material for structure determination. The WFC analyte could potentially be the next intermediate downstream of *N*-Me-DMAT, in which case *easC* would code for the enzyme involved in the third step of ergot alkaloid synthesis. Considering its catalase activity, one proposed function for EasC is detoxification of hydrogen peroxide produced from the oxidative activities of EasE [9], in which case EasC would not act directly on an ergot alkaloid substrate; however, comparison of the alkaloid profiles of WFE and WFC mutants indicates that EasC can act independent of EasE and chemically modifies an ergot alkaloid substrate. In a previous study, *easE* was knocked out in *A. fumigatus*, creating a strain of *A. fumigatus* that lacked *easE* but contained *easC* [9]. *N*-Me-DMAT was detected in cultures of the *easE* knockout strain, but the novel metabolite from the *A. nidulans* WFC strain was not observed. One potential explanation for this apparent discrepancy is that the *A. fumigatus* *easE* knockout mutant contained downstream enzymes in the ergot alkaloid pathway that may have altered its ergot alkaloid profile. Alternatively, the difference between alkaloid profiles of *A. fumigatus easE* knockout and *A. nidulans* WFC could be due to other factors in the species backgrounds in which *N*-Me-DMAT accumulated. It is presently unknown whether the observed WFC analyte is an actual, on-pathway intermediate or whether it is a byproduct of EasC acting out of sequence on a compound that is not ordinarily its substrate. Feeding studies with the unidentified analyte and strains expressing defined sets of ergot alkaloid synthesis genes would be a reasonable approach to test the involvement of this compound in the ergot alkaloid pathway. Again, greater quantities of the analyte would be required for such studies. An *in vitro* approach involving separate and combined enzymes also would help to fully understand the functions of these enzymes. Difficulty with expressing EasE in active form *in vitro* has slowed studies of catalytic function of this enzyme [9]. 

The approach of expressing genes in *A. nidulans* may be applied to other genes in the pathway to study their roles in the biosynthesis of ergot alkaloids (as was recently reviewed by Chiang *et al.* [16] for other fungal secondary metabolite pathways). Heterologous expression in *A. nidulans* also should be applicable to studying genes from ergot alkaloid pathways of other fungi, such as those genes from lineage-specific branches of the pathway. In such cases, it may be necessary to replace the native promoters of those heterologous genes with promoters from *A. fumigatus* *eas* genes or strong *A. nidulans* promoters. 

## 4. Experimental Section

### 4.1. Fungal Strains and Growth Conditions

*Aspergillus fumigatus* isolate Af293, as a source of genomic DNA, was grown in malt extract broth (15 g malt extract/L) overnight at 37 °C. The uridine auxotrophic *A. nidulans* isolate FGSC A767 (Fungal Genetic Stock Center, Kansas City, MO, USA) was grown at 37 °C on SYE medium (20 g/L sucrose, 10 g/L yeast extract, 10 g/L MgSO_4_, 2 mL/L trace element solution [17], 2.2 g/L uracil, 2.2 g/L uridine, with or without 15 g/L agar). For preparation of protoplasts, *A. nidulans* was grown in liquid SYE medium overnight. Transformants were regenerated on selective SYE agar medium which did not contain uracil or uridine and was supplemented with 304 g/L sucrose (to serve as osmoticum as well as carbon source). To test alkaloid accumulation, cultures were grown on SYE agar medium at 37 °C for one week. 

### 4.2. DNA Manipulations

DNA was extracted from *A. fumigatus* according to the protocol described by Richards *et al.* [18]. An 8774-bp fragment containing *dmaW*, *easF*, *easC*, and *easE* was amplified by PCR primed with oligonucleotides primer 1 (5'-TACCTATACCTAATCGAAGCCGCACGCAGTGCACC-3') and primer 2 (5'-GCCGCCCATTCACCAAGATTTTTGCACAAATCTGCG-3'). A 25 μL reaction contained 1× LongAmp Taq reaction buffer, 200 μM of each deoxynucleotide triphosphate, 1 μM of each primer, and 2.5 units LongAmp Taq DNA polymerase (New England BioLabs, Ipswich, MA, USA). The reaction began with an initial denaturing step of 3 min at 94 °C, followed by 35 cycles of 30 s at 94 °C and 10 min at 65 °C, with a final extension of 5 min at 65 °C. 

A 6123-bp fragment containing *dmaW*, *easF*, and *easE* was amplified by PCR primed with oligonucleotides primer 3 (5'-GAGAGCTACTTGACATATTGTGTCGGCAGGTGCGCA-3') and primer 2 (5'-GCCGCCCATTCACCAAGATTTTTGCACAAATCTGCG-3'). The PCR conditions were the same as stated above except the elongation time was 7 min. 

In order to generate transformant WFC (containing *dmaW*, *easF*, and *easC*), two separate DNA fragments were amplified for co-transformation. The first fragment containing *dmaW* and *easC* was primed with primer 1 (5'-TACCTATACCTAATCGAAGCCGCACGCAGTGCACC-3') and primer 5 (5'-CTACTCCATTGTCCTGTGAGTTG-3') which primed amplification of a 4846-bp fragment. The PCR conditions were the same as stated above except the elongation time was 5 min. The second fragment (2943 bp) contained only *easF* and was amplified by PCR primed with oligonucleotides easFF (5'-TCCATTCTTCGCTCGTTCAACCAGCAGG-3') and easFR (5'-CAGGACCTGTACCTAAAGCCTGGTAACC-3') in a 25 μL reaction containing 1× GoTaq Flexi buffer, 200 μM each deoxynucleotide triphosphate, 1.5 mM MgCl_2_, 1 μM of each primer, and 2.5 units of Taq DNA polymerase (Promega, Madison, WI, USA). The reaction began with an initial denaturing step of 3 min at 94 °C, followed by 35 cycles of 30 s at 94 °C, 30 s at 55 °C, and 3 min at 72 °C, with a final extension of 5 min at 72 °C.

PCR products were purified with QIAquick gel extraction kits (Qiagen, Valencia, CA, USA) prior to their inclusion in fungal transformations. The selectable marker pPyrG was obtained from the Fungal Genetics Stock Center and was digested with *Xho*I (New England BioLabs, Ipswich, MA, USA) and purified with QIAquick gel extraction kit prior to transformation.

### 4.3. Fungal Transformation and Screening

Protoplasts of *A. nidulans* FGSC A767 were prepared by incubating overnight cultures with 50 mg driselase (Sigma-Aldrich, St. Louis, MO, USA), which was filter sterilized with a 0.22 μm filter, and 75 mg lysing enzyme (Sigma-Aldrich, St. Louis, MO, USA) in 15 mL of 0.7 M sodium chloride. Protoplasts were purified and co-transformed with PCR product and selectable marker (pPyrG) as described by Coyle *et al.* [19]. Protoplasts were plated on SYE agar medium containing no uracil or uridine. Transformation plates were incubated at 37 °C, and transformants were collected approximately three days after transformation. 

Each transformant was screened by PCR for the presence or absence of *dmaW*, *easF*, *easC*, and *easE*. An internal region of *dmaW* was primed with dmaWF (5'-TTGATCTGGAGTGGTTCCGC-3') and dmaWR (5'-CGTTCATGCCGAAGGTTGTG-3') yielding a 651-bp fragment. Primers for amplifying an internal region of *easE* were easEF (5'-CCAGATACATTGCCATCGCATG-3') and easER (5'-TGTTCCAACTGCTTGGCCAGAT-3'), which primed amplification of a 897-bp fragment. The presence or absence of *easC* was tested by PCR with primers easCF (5'-GAATTCGAGGTATTGATCTCC-3') and easCR (5'-AGCCAGGCAAAGATCCATAGTT-3') which flank a 1036-bp fragment. An internal region of *easF* was primed with easFF (5'-AAGTTGTCGAAGGTCTCACGAA-3') and easFR (5'-GTGATTAGAGATGCTTCTGTC-3'), yielding an 820-bp fragment. Each of these PCRs were 25 μL reactions containing 1× GoTaq Flexi buffer, 200 μM deoxynucleotide triphosphate, 1.5 mM MgCl_2_, 1 μM of each primer, and 2.5 units of Taq DNA polymerase (Promega, Madison, WI, USA). PCR conditions began with an initial denaturing step of 3 min at 94 °C, followed by 35 cycles of 30 s at 94 °C followed by 30 s at 55 °C, and 1 min and 30 s at 72 °C, followed by a final extension of 5 min at 72 °C.

### 4.4. HPLC Analyses

Transformants positive for the presence of the target genes were analyzed by HPLC for the accumulation of chanoclavine-I. Ergot alkaloids were extracted by incubating a spore-rich sample of the colony, containing approximately 1 cm^2^ of culture surface area, in 400 μL of methanol for 30 min and clarifying the extract by centrifugation. Extracts were injected into a C_18_ column (Prodigy 5-μm ODS3 (150 mm by 4.6 mm); Phenomenex, Torrance, CA, USA) and subjected to a multilinear binary gradient from 5% (*v*/*v*) acetonitrile plus 95% (*v*/*v*) aqueous 50 mM ammonium acetate to 75% acetonitrile plus 25% aqueous 50 mM aqueous ammonium acetate at a flow rate 1 mL/min, as previously described [15]. Ergot alkaloids were detected using two fluorescence settings. The first setting had an excitation wavelength of 272 nm and emission wavelength of 372 nm; the second setting was 310 nm (excitation) and 410 nm (emission). Chanoclavine-I (generously provided by Brian Tapper, AgResearch, New Zealand) and *N*-Me-DMAT (gift from Sarah O’Connor, University of East Anglia) were run as standards. Peaks of interest were isolated from the HPLC by collecting the runoff from the discard tube for 30 s after the peak was visualized on the detector. 

### 4.5. LC-MS Analyses

Ergot alkaloid extracts were prepared by washing the surface of a conidiating colony with 2 mL of methanol, rotating the extract at 30 rpm for 30 min, and then clarifying the extract by centrifugation. Clarified extracts were then concentrated to a volume of 50 μL in a speed-vac apparatus, and 10 μL of concentrated extract were injected into the LC-MS. To isolate individual analytes, concentrated extracts were analyzed by fluorescence HPLC, peaks were collected manually, and concentrated to a volume of 50 μL in a speed-vac apparatus prior to LC-MS analysis. Concentrated samples were analyzed on a Finnigan LCQDecaXP plus mass spectrometer equipped with a Surveyor HPLC system. Analytes were separated on a C_18_ column (Phenomenex 4-μm polar-RP (150 mm by 2 mm)), maintained at 30 °C, by combining mobile phases A (5% acetonitrile, 0.1% formic acid) and B (75% acetonitrile, 0.1% formic acid). Initial conditions were 86% A + 14% B, which ramped linearly to 73% A + 27% B at 25 min, 25% A + 75% B at 40 min, and 0% A + 100% B at 42 min. The flow rate was 200 μL/min. Analytes were ionized by electrospray ionization in positive mode and were detected by scanning for ions with a *m/z* between 200–400. 

## 5. Conclusions

Four genes common to all ergot alkaloid synthesis gene clusters—*dmaW*, *easF*, *easE*, and *easC*—are sufficient to direct synthesis of chanoclavine-I in *A. nidulans*. Both *easC* and *easE* are required for the synthesis of chanoclavine-I from *N*-Me-DMAT, but neither alone is sufficient. *Aspergillus nidulans* is a suitable heterologous host for expressing ergot alkaloids synthesis genes. 
